# Estimated Comparative Integration Hotspots Identify Different Behaviors of Retroviral Gene Transfer Vectors

**DOI:** 10.1371/journal.pcbi.1002292

**Published:** 2011-12-01

**Authors:** Alessandro Ambrosi, Ingrid K. Glad, Danilo Pellin, Claudia Cattoglio, Fulvio Mavilio, Clelia Di Serio, Arnoldo Frigessi

**Affiliations:** 1 University Center of Statistics for the Biomedical Sciences, Vita-Salute San Raffaele University, Milan, Italy; 2 Department of Mathematics, University of Oslo, Oslo, Norway; 3 Division of Genetics and Cell Biology, Istituto Scientifico H. San Raffaele, Milan, Italy; 4 Department of Molecular and Cell Biology, University of California at Berkeley, Berkeley, California, United States of America; 5 Center for Regenerative Medicine, University of Modena and Reggio Emilia, Modena, Italy; 6 Department of Biostatistics, University of Oslo, Oslo, Norway; Imperial College London, United Kingdom

## Abstract

Integration of retroviral vectors in the human genome follows non random patterns that favor insertional deregulation of gene expression and may cause risks of insertional mutagenesis when used in clinical gene therapy. Understanding how viral vectors integrate into the human genome is a key issue in predicting these risks. We provide a new statistical method to compare retroviral integration patterns. We identified the positions where vectors derived from the Human Immunodeficiency Virus (HIV) and the Moloney Murine Leukemia Virus (MLV) show different integration behaviors in human hematopoietic progenitor cells. Non-parametric density estimation was used to identify candidate comparative hotspots, which were then tested and ranked. We found 100 significative comparative hotspots, distributed throughout the chromosomes. HIV hotspots were wider and contained more genes than MLV ones. A Gene Ontology analysis of HIV targets showed enrichment of genes involved in antigen processing and presentation, reflecting the high HIV integration frequency observed at the MHC locus on chromosome 6. Four histone modifications/variants had a different mean density in comparative hotspots (H2AZ, H3K4me1, H3K4me3, H3K9me1), while gene expression within the comparative hotspots did not differ from background. These findings suggest the existence of epigenetic or nuclear three-dimensional topology contexts guiding retroviral integration to specific chromosome areas.

## Author Summary

Understanding how retroviral vectors integrate in the human genome is a major safety issue in gene therapy, since a concrete risk of developing tumors associated with the integration process has been observed in several clinical trials. Statistical analyses confirmed the non randomness of the integration. Where and why do virus-specific integrations tend to accumulate in the genome? We compared integration preferences of two retroviral vectors derived from HIV and MLV, which are used in most gene therapy trials for hematological disorders, in their actual clinical targets, *i.e.*, human hematopoietic stem/progenitor cells. We developed a new statistical method to find areas of the genome, called *comparative hotspots*, where integration preferences are significantly different. We modeled the integration process as a stochastic process, so that integration sites are seen as samples from an unknown virus-specific probability density function. Thus, the problem became to identify areas where two empirical density functions differ significantly. The comparison of nonparametric variability bands around the estimated integration densities allowed identifying and ranking candidate comparative hotspots. Results indicated clear differential patterns of integration between HIV and MLV, leading to new hypotheses on the mechanisms governing retroviral integration.

## Introduction

Seminal clinical studies have recently shown that transplantation of stem cells, genetically modified by retroviral vectors, may cure severe genetic diseases such as immunodeficiencies [1], [2], skin adhesion defects [3] and lysosomal storage disorders [4]. Unfortunately, some of these studies also showed the limitations of retroviral gene transfer technology, which may cause severe and sometimes fatal adverse effects. In particular, insertional activation of proto-oncogenes by vectors derived from the Moloney murine leukemia virus (MLV) caused T-cell lymphoproliferative disorders in five patients undergoing gene therapy for X-linked severe combined immunodeficiency [5], [6], and pre-malignant expansion of myeloid progenitors in two patients treated for chronic granulomatous disease [7]. Pre-clinical experiments showed that HIV-derived lentiviral vectors are less likely to cause insertional gene activation than MLV vectors. Most of the studies on retroviral integration preferences, however, have been carried out on cell lines that poorly represent the genomic characteristics of somatic stem cells, or on limited numbers of patient-derived cells. A better understanding of the interactions between retroviral vectors and the genome of clinically relevant target cells may provide a more rational basis for predicting genotoxic risks in clinical gene therapy.

A large number of studies have focused on the molecular mechanisms by which mammalian retroviruses choose their integration sites in the target cell genome. After entering a cell, the retroviral RNA genome is reverse transcribed into double-stranded DNA, and assembled in pre-integration complexes (PICs) containing viral as well as cellular proteins. PICs associate with the host cell chromatin, where the virally encoded integrase mediates proviral insertion in the genomic DNA. Retroviral integration is a non-random process, whereby PICs of different viruses recognize components or features of the host cell chromatin in a specific fashion [8]. The LEDGF/p75 protein has been identified as the main factor tethering HIV PICs to active chromatin [9], while mechanisms underlying integration site selection of other retroviruses remain largely unknown. We recently showed that MLV-derived vectors integrate preferentially in hotspots near genes involved in the control of growth, differentiation and development of hematopoietic cells and flanked by defined subsets of transcription factor binding sites; this suggested that MLV PICs are tethered to transcriptionally active regulatory regions engaged by basal components of the RNA Pol II transcriptional machinery [10], [11]. On the contrary, HIV-derived vectors target expressed genes in their transcribed portions away from regulatory elements, suggesting a different evolutionary strategy for these two viruses.

The molecular basis of retroviral target site selection is still poorly understood. The concept of integration “hotspot” was introduced to describe areas of the genome where integrations accumulate more than expected by chance in the absence of any selection process [10]. Hotspots therefore differ from “common integration sites” (CIS), which were defined as sites recurrently associated with virus-induced malignant expansion [12]. The final goal in finding hotspots is to investigate genomic properties that lead certain areas to “attract” or “refuse” integration. We suggested in previous work that integration preferences are dependent on the intrinsic gene density distribution and on the type of vector [13], [14].

In this paper we develop a statistical methodology to detect “comparative” hotspots, i.e. areas of the genome where integration intensities of MLV and HIV appear to differ. We do not find regions where the viruses prefer to integrate, but where the integration patterns are different. Our approach followed two steps: first candidate comparative hotspots were identified by comparing variability bands around estimated integration intensities along the genome, and then each candidate comparative hotspot was tested in turn. After multiplicity correction we produced a list of 100 comparative hotspots, ready for further biological validation. Our analysis discriminated regions which were targeted by both viruses, most likely on the basis of their accessibility (high content of active genes), from regions specifically that are preferred by either MLV or HIV. We show that HIV and MLV integrate differently in regions spanning 0.2 to >6 Mb in the human genome, with specific patterns. In particular, HIV-specific hotspots are wider and contain a larger number of genes. The preference of HIV or MLV for these regions cannot be explained by the known viral target site selection preferences, or by the expression characteristics of the targeted genes, suggesting the existence of epigenetic or nuclear topology contexts that drive retroviral integration to specific chromosome territories.

## Results

**Figure 1:**
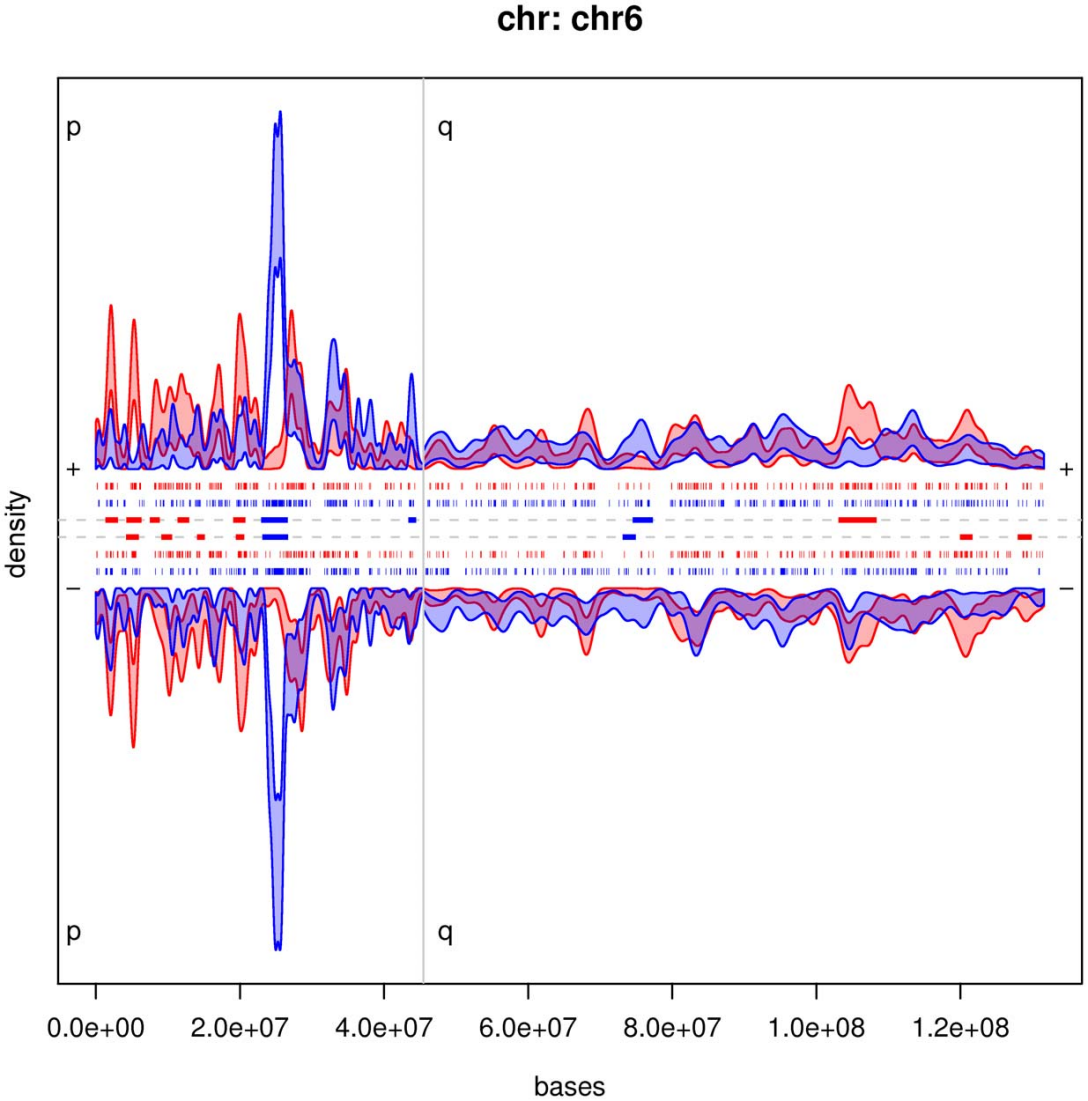
**Integration densities of HIV and MLV in CD34^+^ cells, for chromosome 6.** We analyzed each strand separately: the upper half is the + strand and the lower the - strand. In blue the estimated variability band at level 0.99 for HIV integrations (n = 1629), in red for MLV (n = 1815). Candidate comparative hotspots are plotted in the two central *x*-axes, the color indicating which of the two vectors had stronger integration intensity (HIV: blue; MLV: red). In the other four *x*-axes, each tick represents one integration site, with the same color code. Because of resolution, many ticks fall on the same point and cannot be distinguished.

We developed a new statistical method to compare the integration preferences of distinct retroviral vectors in the human genome and we used it to analyze a collection of ∼30,000 MLV and HIV-vector insertion sites in human CD34^+^ hematopoietic stem-progenitor cells [15]. Figure 1 illustrates how the methodology performs on chromosome 6. We compared the two integration propensities for each arm and strand separately. The blue 99% variability band corresponds to the integration density of HIV, estimated from our data; in red the band for MLV. When the two bands stay apart, one above the other, a candidate comparative hotspot is identified as the segment of such empty intersection. These are depicted as blue and red thick segments in the center of the plot. In chromosome 6 we identified 12 candidate hotspots where MLV shows more integrations than HIV, and 5 candidate hotspots where HIV is dominating. In most of chromosome 6, we found no differences in integration patterns. Note the high peak of integration on the p-arm for HIV, on both strands, corresponding to the MHC locus. Similar plots for all chromosomes are available in supplementary material Figure S1 and Figure S2.

**Figure 2:**
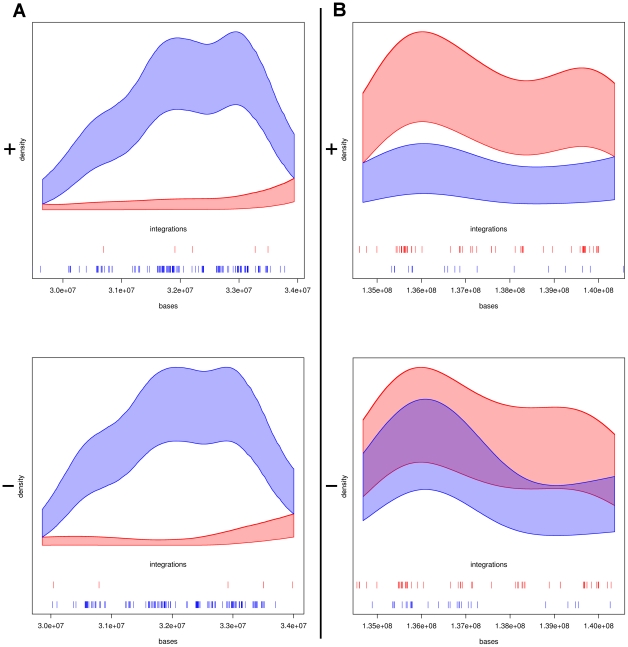
**Two typical situations in comparative hotspots.** In panel A (left side) the bands don't overlap in plus and minus strands, suggesting the presence of two candidate comparative hotspots (hotspots ID: hiv_36 and hiv_40, see supplementary material, Table S1). Differences in integration densities in one versus the other strand may reflect a preferential integration orientation at that particular locus. In panel B (right side) the bands don't overlap in the plus strand (upper panel) whereas on the minus strand they do (lower panel), suggesting only one candidate (hotspot ID: mlv_43, see supplementary material, Table S1). These examples are taken from chromosome 6.

The panels of Figure 2 show two typical situations in detail. The panel A (left side) from the HIV HLA locus in chromosome 6, arm p; upper panel refers to strand+, lower panel refers to strand -. We see how the estimated variability bands, around the non-parametrically estimated integration densities, are clearly apart from each other. The bands overlap at both ends of the comparative hotspot, which is therefore well defined. The width of the bands describes the statistical uncertainty attached to the estimated densities: in both cases the MLV bands are quite thin, as there are a total large number of integrations. The bands for HIV are larger; the exact density function is difficult to estimate with limited sample size. Despite the uncertainty, the candidate hotspot in panel A is clearly identified. The panels B of Figure 2 show a candidate comparative hotspot in the plus strand of chromosome 6, arm q, which has no corresponding in the minus strand. In other locations of the genome, the two bands often overlap simply due to lack of data, rather than because the two vectors are equally distributed. This indicates that our method will leave undiscovered comparative hotspots (false negatives). Not all the candidate comparative hotspots that we identified were clearly distinguishable.

Our analysis led to 256 candidate comparative hotspots on all chromosomes (see, supplementary material, Table S1). Each candidate comparative hotspot was then tested individually. We computed odds ratios, between HIV and MLV odds of integrations in each hotspot, and tested the null hypothesis that the odds ratio is one. P-values were then corrected for multiple testing. This reduced the number of significative comparative hotspot to 100, reported in Table 1.

**Table 1:** Comparative Hotspots.

virus	chr	strand	start	end	length	OR	adjusted-p	# genes
hiv	chr11	+	63052973	68240744	5187771	6.73	1.16e-052	177
hiv	chr6	−	29857643	34003291	4145648	25.59	7.53e-046	171
hiv	chr16	−	0	3573133	3573133	9.82	1.85e-045	171
hiv	chr11	−	63408683	68252636	4843953	5.23	6.59e-045	169
hiv	chr6	+	29653216	33939640	4286424	31.23	2.42e-043	179
hiv	chr16	+	0	3106569	3106569	13.06	3.32e-042	153
hiv	chr1	+	0	4770330	4770330	14.32	1.66e-027	89
hiv	chr3	−	46696908	53554160	6857252	4.03	1.58e-025	159
hiv	chr17	−	70567573	74031223	3463650	4.35	1.14e-024	81
hiv	chr17	+	77083925	78700791	1616866	8.53	2.45e-024	56
hiv	chr9	+	136302969	140273252	3970283	7.96	4.03e-023	97
hiv	chr1	−	151528646	154707353	3178707	4.77	4.52e-020	114
hiv	chr9	−	135483396	140273252	4789856	6.70	1.62e-019	105
hiv	chr3	+	46805137	51013082	4207945	3.78	1.18e-014	101
hiv	chr1	+	152393200	155051471	2658271	3.61	1.36e-014	97
hiv	chr8	+	143266499	146274826	3008327	5.29	1.76e-013	94
hiv	chr17	−	76476613	78537508	2060895	4.42	1.85e-013	67
hiv	chr2	−	26376098	28509807	2133709	5.32	4.64e-013	51
hiv	chr8	−	142937251	146274826	3337575	5.58	7.07e-013	94
hiv	chr17	+	71000437	72361727	1361290	5.03	2.53e-012	50
mlv	chr18	+	71458533	76117153	4658620	14.38	1.65e-011	17
mlv	chr21	−	38477753	39470763	993010	23.96	1.13e-010	5
hiv	chr22	+	48573053	49691432	1118379	6.34	8.99e-010	38
hiv	chr20	−	60274607	62435964	2161357	6.34	8.99e-010	60
mlv	chr17	+	51301476	53344785	2043309	15.10	5.64e-009	12
hiv	chr4	−	0	3791201	3791201	4.67	1.23e-008	61
hiv	chr16	+	86724460	88827254	2102794	4.08	3.24e-008	45
mlv	chr20	+	51190248	52356969	1166721	13.94	5.88e-008	6
hiv	chr19	+	54403063	55308772	905709	5.97	7.82e-008	47
mlv	chr12	+	115324321	117477353	2153032	10.89	1.10e-007	16
mlv	chr6	−	5956453	7313927	1357474	33.96	1.20e-007	6
hiv	chr2	+	26617223	28096371	1479148	3.50	1.34e-007	47
hiv	chr19	−	54293977	55393350	1099373	5.65	4.17e-007	54
hiv	chr22	−	48767773	49691432	923659	4.40	4.85e-007	33
hiv	chr2	−	185808293	188459285	2650992	8.25	5.51e-007	7
mlv	chr18	−	71648647	73379689	1731042	10.02	1.04e-006	5
hiv	chr12	+	47052128	48977359	1925231	4.53	1.39e-006	53
hiv	chr15	+	38942654	41945536	3002882	3.80	2.97e-006	61
mlv	chr20	−	51173685	52422011	1248326	9.58	3.17e-006	6
mlv	chr3	+	70940451	72487606	1547155	9.36	5.55e-006	4

List of the 40 top hotspots for which the p-value (Bonferroni-Holm adjusted 41) of the odds ratio (OR) being equal to one, was below 0.05. The first column indicates which virus had most integrations. Columns 2–5 locates the hotspot on its chromosome. Column 6 contains the width of the hotspot (min: 211313 bp, max: 6857252). The OR (column 7) was always defined to be larger than 1 (min: 2.24, max: Inf). The adjusted p-values are in column 8. The number (#) of genes included in each hotspot is in the last column (range: 1 to 179). Full table is available in supplementary material Table S1.

doi:10.1371/journal.pcbi.1002292.t001

**Figure 3:**
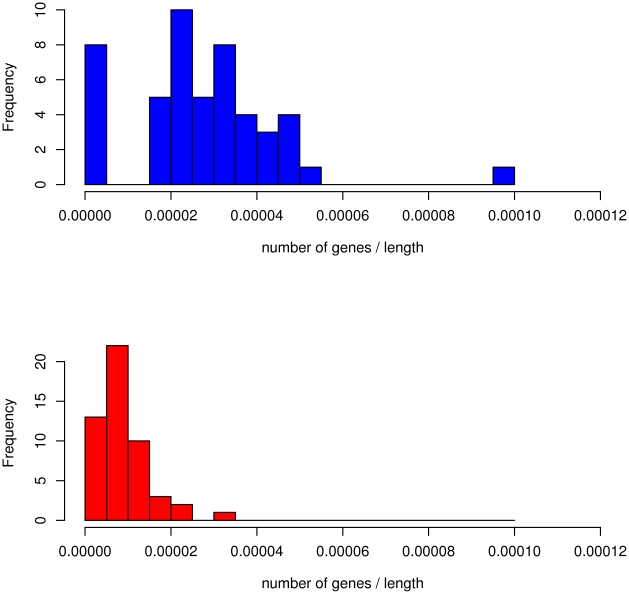
**Gene density of HIV and MLV comparative hotspots.** Histogram of the number of target genes per hotspot normalized by hotspot length in HIV (blue bars, upper panel) and MLV (red bars, lower panel).

The length of the hotspots varies between ca. 200,000 bp and 7,000,000 bp, but most are longer than 10^6^ bp. They include between 1 and 179 genes. Of the 100 significative comparative hotspots, 49 have a higher density of HIV integrations (lengths ranging between 378,200 and 6,857,000 bp; median: 2,651,000 bp, 2,027 unique target genes) while 51 contain a higher MLV density (lengths ranging between 211,300 and 6,021,000 bp; median: 1,319,000 bp, 475 unique target genes). The median length of MLV hotspots is about the half of the median length of HIV hotspots with a significative difference (p-value: 2.108•10^−06^; Mann-Whitney test, p-values computed by permutations). The wideness of HIV hotspots only partially accounts for the higher number of target genes compared to MLV (2,027 *vs.* 475), as shown by plotting the number of targets per hotspot normalized by the hotspot length (see Figure 3: p-value = 1.953*10^−8^, Mann-Whitney test, p-values computed by permutations). This hints at gene density as a critical parameter for HIV integration site selection and is in accordance with the recent finding that MLV integration is associated to transcription regulatory regions rather than to genes 11,15,16.

To investigate the categories of genes preferentially targeted by the comparative hotspots we performed a Gene Ontology (GO) classification of HIV and MLV target genes (supplementary material, Table S2). Among the 2,027 genes in the comparative hotspots with HIV preference, the analysis showed a significant enrichment over the background (0.005<p-values<0.05, Fischer's exact test with Bonferroni correction for multiple testing) for genes involved in antigen processing and presentation, and in hormone nuclear receptor activity. Remarkably, both GO terms exclusively contained genes located in the MHC locus on chromosome 6 (highest peak in Figure 1). Among the 475 MLV targets, genes participating in adaptive immune response, signal transduction, and regulation of biological processes were over-represented (0.005<p-values<0.05). Differently from HIV targets, these genes did not belong to the same chromosomal region.

The annotation of oncogenes 17 (Sanger Cancer Gene Census, http://www.sanger.ac.uk/genetics/CGP/Census) incorporated into comparative hotspots (see the full gene Table S1 in supplementary material) did not reveal a significant difference in the targeting frequency between the two vectors, both when considering all genes (0.045 for HIV *vs.* 0.050 for MLV, p-value: 0.4109) or the sole genes in the 100 significative comparative hotspots (0.047 for HIV *vs.* 0.054 for MLV, p-value: 0.06707). We next investigated the relation between gene expressions and comparative integration hotspots. We compared the frequency of expressed genes belonging to comparative integration hotspots with the frequency of transcribed genes located elsewhere in the genome. After multiple testing corrections, we found just one hotspot with increased presence of expressed genes with respect to the genomic baseline (hiv_55, adjusted p-value 0.02800; see Table S1, supplementary material for full results). Three comparative hotspots, all with higher MLV density (mlv_50, mlv_51, mlv_124, p-values: 0.00075, 00.00197, 0.00778 respectively), showed instead a reduced presence of expressed genes.

Since there is strong evidence of association between integration sites and specific histone modifications 18,19,20, we also investigated the histone methylation 21 density in comparative hotspots, defined as the methylation intensities (i.e., the number of ChIP-seq reads) in each comparative hotspot, divided by the hotspot length; the same was done for the histone variant H2A.Z. We compared the mean density of histone modifications associated to transcription or heterochromatin in HIV vs. MLV hotspots using the Welch statistic test, which does not assume the same variance for the two groups, with p-values computed by permutations. After adjustment for multiplicity (Bonferroni-Holm) three methylations and the one histone variant analyzed were found to have different mean density in HIV vs. MLV hotspots (H3K4me1, H3K4me3, H3K9me1, H2AZ; adjusted p-values: 0.000096, 0.000010, 0.020986, 0.000018 respectively). Results are summarized in table 2.

**Table 2:** Density of histone modifications in comparative hotspots.

methylation	HIV		MLV		p	adjusted-p
	mean	SD	mean	SD		
H3K27me3	0.002805	0.001179	0.003140	0.001585	0.069324	0.347210
**H2AZ**	0.003625	0.001441	0.004494	0.001444	2e-06	**0.000018**
H3K27me1	0.003399	0.000889	0.003774	0.001235	0.008568	0.050412
H3K36me3	0.006188	0.005843	0.005998	0.003063	0.766001	1
**H3K4me1**	0.002469	0.001630	0.003486	0.001823	6e-06	**0.000096**
**H3K4me3**	0.001617	0.001179	0.001078	0.000494	1e-06	**0.000010**
**H3K9me1**	0.006676	0.004553	0.008585	0.005302	0.00297	**0.020986**
H3K9me3	0.003696	0.003790	0.003555	0.001467	0.728885	1
H4K20me1	0.008214	0.005921	0.007407	0.004374	0.212219	0.846512
PolII	0.001876	0.000829	0.002016	0.001054	0.263484	0.846512

Columns 2 and 3 report the mean density of modifications and relative standard deviation in comparative hotspots with HIV abundance. Columns 4–5 are mean density and standard deviation in MLV preferred hotspots. The p-values for the equality of the means are in column 5 (Mann-Whithney test statistics, p-values computed by permutations) and adjusted p-values in column 6 (Bonferroni-Holm method). In bold, methylations/histone variants with significative difference.

doi:10.1371/journal.pcbi.1002292.t002

**Figure 4:**
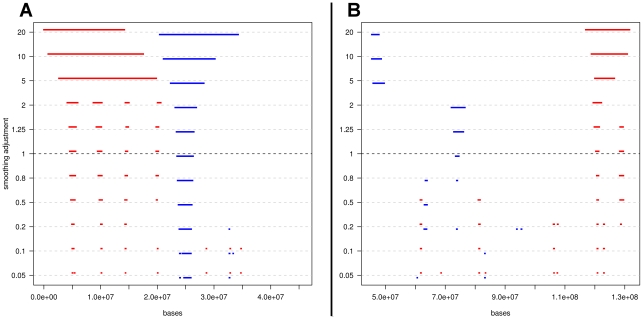
**Robustness plots.** Panel A: chromosome 6, arm p; strand -. Panel B: chromosome 6, arm q; strand -. Here we can observe how hotspots would change in length and location if we were to use different smoothing parameters. Most importantly, we see that the hotspots identified at level 1, corresponding to our choice of the smoothing parameters, persist at slightly larger and smaller values, confirming their validity. At smaller levels of smoothing many spurious hotspots appear, of very short length. There is no support from the data for these, as they either disappear for more smoothing or they merge into larger and more robust segments. Large smoothing either impairs the creation of hotspots (as bands tend to become large and flat) or they deliver very large hotspots, which are difficult to interpret biologically.

The construction of the variability bands of an integration density depends on a design smoothing parameter, as described in the Materials and Methods section. The choice of such smoothing parameters controls the regularity of the variability bands and therefore had an effect on the comparison. We estimated the smoothing parameters in an optimal fashion (in correspondence to which results were reported), but also studied robustness of the hotspots by varying them systematically. We systematically checked if a comparative hotspot would have persisted for larger and smaller smoothing parameters. Figure 4 shows the results of such a sensitivity study for two strands of chromosome 6. The middle line, corresponding to 1, shows the hotspot identified by the two optimal smoothing parameters, while above and below that we see how hotspots would grow and shrink by increasing and reducing the smoothing level. It is important that the chosen segments at level 1 continue to appear for values just above and under, as happened systematically. This visual inspection strengthens the validity of the way we chose the smoothing parameters. See Methods for more details and supplementary material Figures S3, S4, S5, S6, S7 and S8 for robustness plots for all chromosomes.

## Discussion

Integration of MLV-derived retroviral vectors may have significant consequences on gene expression and homeostasis of transduced and transplanted target cells, particularly in the hematopoietic system. The enhancer activity of the MLV LTRs may de-regulate proto-oncogenes, and cause pre-neoplastic clonal expansion 7,22, leukemic transformation without clonal expansion 5,6,23, or no apparent adverse effect 1 depending on the disease context and a number of still ill-defined factors. Integration sites can be used as markers of clonality to study the clonal dynamics of transduced cells *in vivo*, and provide important clues to predict the potential genotoxicity of MLV integration in a specific cell or disease context 23,24,25,26,27. We used LM-PCR and pyrosequencing to derive high-definition maps of MLV and HIV integration sites in the genome of human CD34^+^ hematopoietic progenitors. As previously reported 14, MLV integrations were clustered around gene regulatory elements (promoters, enhancers, evolutionarily conserved non-coding regions) bearing epigenetic marks of active transcription (H3K4me1, H3K4me2, H3K4me3, H3K9Ac) and specialized chromatin configurations (H2A.Z). On the contrary, HIV integrations occurred away from regulatory elements, and are associated with histone modification enriched in the body of transcribed genes (H3K36me3 and H2BK5me1). In both cases, statistical analysis identified hotspots of clustered integrations with strong correlation with transcriptional activity, using random integration datasets as controls.

In this study, we identify broad areas of the genome where HIV and MLV integrate differently; therefore it was not expected to find comparative hotspots in areas of high gene expression. This is in accordance with the fact that a single hotspot showed an increased expression level with respect to the rest of the genome. We used non-parametric density estimation and variability bands to identify regions of the genome as candidate comparative, i.e., virus-specific, hotspots. Thereafter, these were tested for significance. The first step delivers a series of bins, of variable length, were the two integration frequencies appear to be different. This strategy is more effective than binning the chromosome with equal size bins, since some of them might not be large enough to contain enough integrations. An optimal bin size algorithm, producing a constant bun size, would easily divide a chromosome in a dozen bins, which would be too large to be of practical interest as candidate hotspots. Our approach generates a list of bins of variable and adaptive length, only in areas of interest. Interestingly, this analysis identified large genomic regions (0.2 to >6 Mb in length) rather than local (<100 kb) hotspots. Most genomic regions are targeted by both virus types, most likely because they contain a high proportion of active genes and regulatory elements. Some regions, however, are targeted by either virus in a specific fashion, where HIV-specific hotspots tend to be larger in size and to contain more genes. The expression and gene ontology characteristics of the genes contained in MLV and HIV-specific regions, however, were comparable, and there are no obvious characteristics that would predict such a striking virus-specific preference. While MLV-specific regions are enriched for histone modifications/variants correlated with active regulatory regions (H3K4me1, H2A.Z), HIV-specific regions have a higher density of H3K4me3, associated to active transcription start sites. Although counterintuitive, given the well-known MLV preference for transcription start sites, this might be simply explained by the higher gene content of HIV-specific hotspots. Unfortunately, genomic distribution of HIV tethering factors, such as LEDGF/p75, is not known, particularly for hematopoietic progenitors, and it is therefore impossible to test whether high protein concentration in specific chromosomal region may explain the HIV-specific preferences.

Interestingly, we found a significant comparative hotspot spanning the entire MHC locus on chromosome 6 (from the MHC class I to the extended MHC class II subregions 28) with increased HIV, but not MLV, integration propensity. Importantly, a gene-centric hotspot definition would have failed to detect this locus, since in this particular case intergenic regions rather than single genes are highly targeted by HIV.

Large, virus-specific hotspots may suggest that tethering of PICs to chromatin favors relatively wide chromosomal territories independently from their content or local concentration of “attractive” features, such as GC content of DNA, binding of factors or transcriptional complexes, nucleosome density or epigenetic marks. This type of preference may instead reflect larger scale, nuclear topology factors that make these regions more accessible to one or another virus type. The modalities by which HIV and MLV access target cell chromatin, may be a critical factor underlying these preferences. MLV is incapable of entering intact nuclei and requires cell division in order to integrate, while HIV is actively imported in interphase nuclei through the nuclear pores. MLV and HIV PICs therefore “see” chromatin in different phases of the cell cycle, and may have access to different regions simply because they are differently exposed. Recent studies showed that alterations in the nuclear pore architecture impairs HIV nuclear import and impacts on integration efficiency, suggesting that access to chromatin is mediated by the nuclear pore and may be a critical component of target site selection 29,30. The HIV-specific hot regions identified in this study may therefore reflect the chromatin organization in the vicinity of the nuclear pore. Studies are in progress to test this hypothesis in clinically relevant target cells.

## Materials and Methods

### Integration Within Cd34^+^ Cells

We worked with a previously published collection of 28,382 HIV and 32,631 MLV retroviral integration sites isolated by linker-mediated PCR (LM-PCR) and pyrosequenced by GS-FLX Genome Sequencer (Roche/454 Life Sciences, Branford, CT) from cord blood-derived human CD34^+^ hematopoietic stem-progenitor cells 15. The bioinformatics pipeline used to process crude MLV and HIV sequence reads was previously described 15. Briefly, valid reads 20-bp or longer were used to generate a non-redundant dataset using the nrdb tool (available at http://www.advbiocomp.com/blast.html in the AB-BLAST software package). Non-perfectly redundant reads were than mapped onto the human genome, requiring the alignment to start within the first three nucleotides and to possess a minimum of 90% identity. Sequences were discarded when mapping to multiple sites if they had more than one match on the human genome differing in identity less than 2%. Overall valid sequence recovery was similar between MLV and HIV (13.3% and 17.3%, respectively). The expression profile of CD34^+^ cells was determined by microarray analysis of cytokine-activated cells from three independent umbilical cords. RNA was extracted from 1–2×10^6^ cells, transcribed into biotinylated cRNA and hybridized to Affymetrix HG-U133A plus 2.0 Gene Chip arrays.

### Functional Clustering Analysis

Functional clustering of target genes was performed by the DAVID 2.0 Functional Annotation Tool and EASE score, as previously described 10. GO categories were considered over-represented when yielding an EASE score <0.05, after Bonferroni-Holm correction for multiple testing.

### Blind Regions

**Figure 5:**
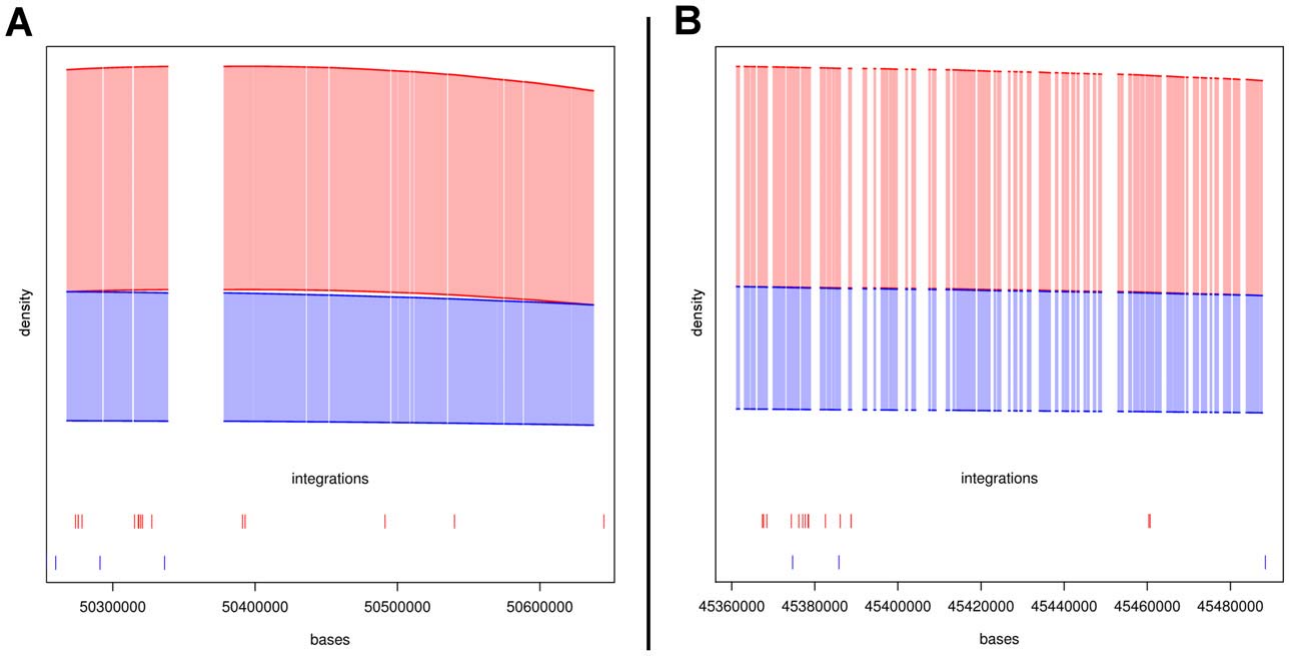
**Blind regions.** These plots illustrate the presence of blind regions, which are scattered over the genome and usually short, but occasionally also of appreciable length (panel A: mlv_53, chromosome 7, arm p strand -; panel B: mlv_147, chromosome 22 arm q, strand +). Not all the candidate comparative hotspots that we identified were clearly distinguishable, see for example panel A.

Certain areas of the genome cannot be scanned in order to investigate the presence of integrations. This is mainly due to two reasons: genome mappability and the presence of what we call “blind regions”. Although extremely critical in determining the randomness of single integration patterns, genome mappability was not a concern in our comparative study, since only unequivocally mapping reads were considered, for the comparison of MLV and HIV integration patterns (*i.e.*, the mappability bias, if any, was the same for the two vectors). Blind regions instead derive from the use of restriction enzymes and size-selection during the integration library preparation, and represent portions of the genome that are scarcely accessible to detection due to their distance to the closest 3′ restriction site (Figure 5). Specifically, if this distance is shorter than the sensibility of alignment programs, in terms of minimum length of the processable sequence, integration is not identifiable. For example, if a viral vector integrated 10 bps far from the closest 3′ cut sequence, then from the sequencing platform we obtained a 10 bps sequence, that for most of alignment program is not processable. We used Blat 31 which has minimum sequence length of 20 nt. On the other hand, the size-fractionation step only includes fragments <500 nt, this being the maximum estimated length for efficient 454 bead loading (see supplemental methods in 10). Therefore, integrations with a distance to the closest 3′ restriction enzyme site of, for example, 600 bps, would not be detected. These blind regions need to be excluded from further analysis, as it was impossible to determine accurately integration frequencies occurring therein. We first identified these blind regions by looking for the position of restriction sequences over the whole genome. We then cut off the blind regions. When performing density estimation, we skipped blind regions and connected together successive non-blind parts. We assumed smoothness of the density at mending points, as the blind regions were comparably short. Once hotspots were found, the blind areas were placed back in the original topology. Integration analysis was performed separately for each chromosomal arm, so that it was not affected by the centromere, which is a giant blind region. Furthermore, we studied separately each strands, since blind regions are strand specific. The presence of blind regions due to the restriction enzyme digestion is known. It has been shown that the “invisible” portion of the genome is substantially affected by the use of different and/or multiple restriction enzymes 32. We also found the percentage of blind regions to be very significant, ranging from 10% up to 40% of the length of the chromosome. For example, 30.5% of chromosome 1 was blind (total length 247.249.719 bp); see supplementary material Table S1 for percentages for all chromosomes. Blind regions were identified by means of a custom R-script (R ver 2.10 33 and Bioconductor 34) which searched for the TTAA sequences (MseI) on the Hg18 UCSC genome. Once occurrences were identified, blind regions were estimated as follows: from TTAA to 20 bp downstream (due to algorithm limitation) and from 500 bp downstream to the consecutive restriction site (due to deep sequencing platform limitation).

### Statistics

The integration dynamics was modelled as a stochastic process, where integration points were considered as samples from an unknown density function on the region of study *D*. We assumed that each integration was independent of any other. Each virus was considered as a random variable *v* with its own unknown probability density function . Comparing integration preferences of two viruses *v_1_* and *v_2_* was then turned into the statistical problem of comparing two unknown densities and , defined on the same genomic range *D*, based on an independent and identically distributed sample from each of the two densities. The samples were allowed to have different sample size. Our approach was fully nonparametric and led to candidate comparative hotspots, which were then individually tested. Specifically, non-parametric kernel density estimation with Gaussian kernels was used 35. In a basepair , the estimated density , based on the sample in *D*, is given by the kernel density estimatorwhere *K*(·) is the kernel and *h*>0 is the smoothing parameter (bandwidth). We used the Gaussian kernelNotice the scaling of the kernel with *h*, which controls how much weight each integration *x_i_* has in the estimate at a basepair *x*. We performed a small approximation, as the kernel should integrate to 1 over *D*, and the domain *D* is discrete. However, the resolution at basepair level of the chromosome arms is extremely high, so that the effect of this was negligible.

We wished to construct simultaneous confidence bands (at 0.99 level, say) for the two densities to be compared, in order to identify areas (if any) where the confidence bands did not overlap: in such segments of *D*, one density must clearly be below the other. However, such confidence bands depend on the second derivative of the unknown density, controlling both bias and variance; approximations are available only in some special cases under very strong conditions. We instead calculated pointwise variability bands around the estimated densities, where the variation in the density estimates were taken into account, but the bias was ignored. The segments of the chromosome *D* where the two variability bands had empty intersection were considered as candidate comparative hotspots. The 0.99 variability band for the estimated density was computed 36 starting with the Taylor expansionwhereis the integral of the squared kernel function and n is the sample size. The root transform allowed obtaining an approximation of the variance which was independent from the unknown density. Therefore, on the square root scale, a level error band could be computed, using the half widtharound the squared root of the estimate, where is the quantile of the normal standard distribution. Then, as in 36, the edges of this band were transformed back to the original scale aswhere the lower bound is set to zero if it took a negative value. We used .

This is not a confidence band and there is no nominal coverage probability. The effect of the bias is to diminish modes and fill valleys, as it depends on the curvature of (and on the bandwidth), see 36. Variability bands of this type were computed for both densities. Typically, a detected candidate comparative hotspot (where the two variability bands had empty intersection) resulted from a pronounced peak in one density and a valley or flat area in the other. In these situations, adjusting for the bias would have strengthened further the indication of a hotspot. On the other hand, the absence of bias adjustment could in some special situations hide a difference. This indicates that in most cases we have identified candidate comparative hotspots conservatively.

We compared the two pointwise variability bands at level , one for each virus, to detect where the bands did not overlap. These segments in *D* were considered as candidate comparative hotspots. This approach is different from 37, where bins are decided in advance, instead than being data-driven.

Though the band was computed pointwise, it inherited smoothness from the smooth density estimate around which it was built. For computational efficiency, the density was estimated on a grid of points, which were then interpolated with a spline function 38. We did not implement any particular boundary control at the border of the chromosome arm not flanking the centromere.

The choice of the smoothing parameters *h*_1_ and *h*_2_, one for each viral integration density, is important: too much smoothing would flatten the kernel estimates, hiding every difference; too little smoothing would lead to a too rich and fragmented identification of comparative hotspots, with very high false positive findings. Our choice was to perform an automatic and optimal choice of the smoothing parameter for each density and then study how results would change when this value was perturbed in both directions, towards more and towards less smoothing. We chose the optimal smoothing parameters, *h*_opt_, one for each density, using unbiased cross-validation 39. Briefly, *h*_opt_ is chosen to minimize the measure of closeness of to given by the Integrated Squared Errorthrough a least square, leave-one-out crossvalidation criterion. For this purpose we minimized the estimate of the first two terms of the ISE (the last term does not depend on *h*) given bywhere denotes the kernel estimator constructed from the data without the observation . See 39,40 for more details. In order to test sensitivity of results with respect to the choice of *h*, we reparameterized the smoothing parameter as *h* = *h*_opt_ *s*, where the sensitivity factor *s* was left to vary in [0.05, 20]. We then repeated the comparison of the variability bands for the two viral integration densities, using the crossvalidated optimal smoothing parameter for each virus, adjusted with the same *s*. We plotted the comparative hotspots while varying *s*, to see the robustness of each hotspot, as in Figure 4.

Candidate comparative hotspots were then tested individually, to confirm (or not) that the integration propensities of the two viruses were significantly different. As many comparisons were performed, multiple testing correction was done. We computed the odds ratio of the two integration intensities, one for each virus, for each candidate hotspot aswhen HIV had a higher density and the inverse of it when the MLV density was higher instead. Here is the number of integration of HIV falling inside the candidate hotspot *H*, is the number of integration outside hotspot *H*, and similarly for MLV. We computed 0.95 confidence intervals for this odds ratio and tested the null hypothesis that the odd ratio is 1. We used the Fisher exact test. Raw p-values were then corrected for multiple testing by Bonferroni-Holm 41. All computations and analyses were performed in R and Bioconductor environment 33,34.

## Supporting Information

Figure S1 **Integration densities of HIV and MLV in CD34^+^ cells, for chromosomes chr1, chr2, chr3, chr4, chr5, chr6, chr7, chr8, chr9, chr10, chr11 and chr12.** We analyzed each strand separately: the upper half is the+strand and the lower the−strand. In blue the estimated variability band at level 0.99 for HIV integrations, in red for MLV. Candidate comparative hotspots are plotted in the two central *x*-axes, the color indicating which of the two vectors had stronger integration intensity (HIV: blue; MLV: red). In the other four *x*-axes, each tick represents one integration site, with the same color code. Because of resolution, many ticks fall on the same point and cannot be distinguished.
(TIFF)

Figure S2 **Integration densities of HIV and MLV in CD34^+^ cells, for chromosomes chr13, chr14, chr15, chr16, chr17, chr18, chr19, chr20, chr21, chr22, chrX and chrY.** We analyzed each strand separately: the upper half is the+strand and the lower the−strand. In blue the estimated variability band at level 0.99 for HIV integrations, in red for MLV. Candidate comparative hotspots are plotted in the two central *x*-axes, the color indicating which of the two vectors had stronger integration intensity (HIV: blue; MLV: red). In the other four *x*-axes, each tick represents one integration site, with the same color code. Because of resolution, many ticks fall on the same point and cannot be distinguished. Since no integration was found in p-arm of chromosomes chr13, chr14, chr15, chr21 and chr22, in such cases only the q-arm was plotted.
(TIFF)

Figure S3 **Robustness plots for all hotspots, chromosomes chr1, chr2, chr3 and chr4.** The name of each figure identifies the chromosome and the arm and strand. In these figures we can observe how hotspots would change in length and location if we were to use different smoothing parameters. Most importantly, we see that the hotspots identified at level 1, corresponding to our choice of the smoothing parameters, persist at slightly larger and smaller values, confirming their validity. At smaller levels of smoothing many spurious hotspots appear, of very short length. There is no support from the data for these, as they either disappear for more smoothing or they merge into larger and more robust segments.
(TIFF)

Figure S4 **Robustness plots for all hotspots, chromosomes chr5, chr6, chr7 and chr8.** The name of each figure identifies the chromosome and the arm and strand. In these figures we can observe how hotspots would change in length and location if we were to use different smoothing parameters. Most importantly, we see that the hotspots identified at level 1, corresponding to our choice of the smoothing parameters, persist at slightly larger and smaller values, confirming their validity. At smaller levels of smoothing many spurious hotspots appear, of very short length. There is no support from the data for these, as they either disappear for more smoothing or they merge into larger and more robust segments.
(TIFF)

Figure S5 **Robustness plots for all hotspots, chromosomes chr9, chr10, chr11 and chr12.** The name of each figure identifies the chromosome and the arm and strand. In these figures we can observe how hotspots would change in length and location if we were to use different smoothing parameters. Most importantly, we see that the hotspots identified at level 1, corresponding to our choice of the smoothing parameters, persist at slightly larger and smaller values, confirming their validity. At smaller levels of smoothing many spurious hotspots appear, of very short length. There is no support from the data for these, as they either disappear for more smoothing or they merge into larger and more robust segments.
(TIFF)

Figure S6 **Robustness plots for all hotspots, chromosomes chr13, chr14, chr15 chr16 and chr17.** The name of each figure identifies the chromosome and the arm and strand. In these figures we can observe how hotspots would change in length and location if we were to use different smoothing parameters. Most importantly, we see that the hotspots identified at level 1, corresponding to our choice of the smoothing parameters, persist at slightly larger and smaller values, confirming their validity. At smaller levels of smoothing many spurious hotspots appear, of very short length. There is no support from the data for these, as they either disappear for more smoothing or they merge into larger and more robust segments. Since no integration was found in p-arm of chromosomes chr13, chr14 and chr15 in such cases only the q-arm was plotted.
(TIFF)

Figure S7 **Robustness plots for all hotspots, chromosomes chr18, chr19, chr20, chr21 and 22.** The name of each figure identifies the chromosome and the arm and strand. In these figures we can observe how hotspots would change in length and location if we were to use different smoothing parameters. Most importantly, we see that the hotspots identified at level 1, corresponding to our choice of the smoothing parameters, persist at slightly larger and smaller values, confirming their validity. At smaller levels of smoothing many spurious hotspots appear, of very short length. There is no support from the data for these, as they either disappear for more smoothing or they merge into larger and more robust segments. Since no integration was found in p-arm of chromosomes chr21 and 22 in such case only the q-arm was plotted.
(TIFF)

Figure S8 **Robustness plots for all hotspots, chromosomes chrX and chrY.** The name of each figure identifies the chromosome and the arm and strand. In these figures we can observe how hotspots would change in length and location if we were to use different smoothing parameters. Most importantly, we see that the hotspots identified at level 1, corresponding to our choice of the smoothing parameters, persist at slightly larger and smaller values, confirming their validity. At smaller levels of smoothing many spurious hotspots appear, of very short length. There is no support from the data for these, as they either disappear for more smoothing or they merge into larger and more robust segments.
(TIFF)

Table S1 **Comparative Hotspots.** List of the hotspots. The first column indicates the hotspot ID. Column 4 shows which virus had most integrations. Columns 2,3,5,6,7 locates the hotspot on its chromosome. Column 8 and 9 contain the number of integrations. Column 10 contains the width of the hotspot. The OR (column 11) was always defined to be larger than 1. The confidence interval, raw p-values and adjusted p-values are in columns 12–15. The number of genes included in each hotspot is in column 16. The Proto Oncogenes founded in each hotspot is in columns 17. The number of Proto Oncogenes and their density with respect the number of genes in each hotspots are reported in columns 18–19. Number of present and absent genes are in columns 20–21. OR of the present vs absent genes, OR confidence interval, raw p-value and adjusted p-values are reported in columns 22–26.
(XLS)

Table S2 **Gene Ontology (GO) analysis of genes targeted by HIV and MLV comparative hotspots.** 2027 and 475 genes targeted by HIV and MLV comparative hotspots were analyzed by the DAVID Functional Annotation tool 1,2, using the Human Genome as a background population. The table summarizes the significantly over-represented GO categories (GO terms) in the two datasets, after Bonferroni correction for multiple testing. The number of genes included in each GO category is specified (Count), together with their percentage (%) with respect to the total number of genes in the list (List Total) and the fold enrichment over the background. The GO class to which each category belongs is also given (BP: biological process, MF: molecular function, CC: cellular compartment).
(DOC)
