# Supplementary figures and images for: Estimated Comparative Integration Hotspots Identify Different Behaviors of Retroviral Gene Transfer Vectors
Source: PLoS Comput Biol. 2011 Dec 1;7(12):e1002292. doi: 10.1371/journal.pcbi.1002292 (PMC3228801; doi:10.1371/journal.pcbi.1002292)

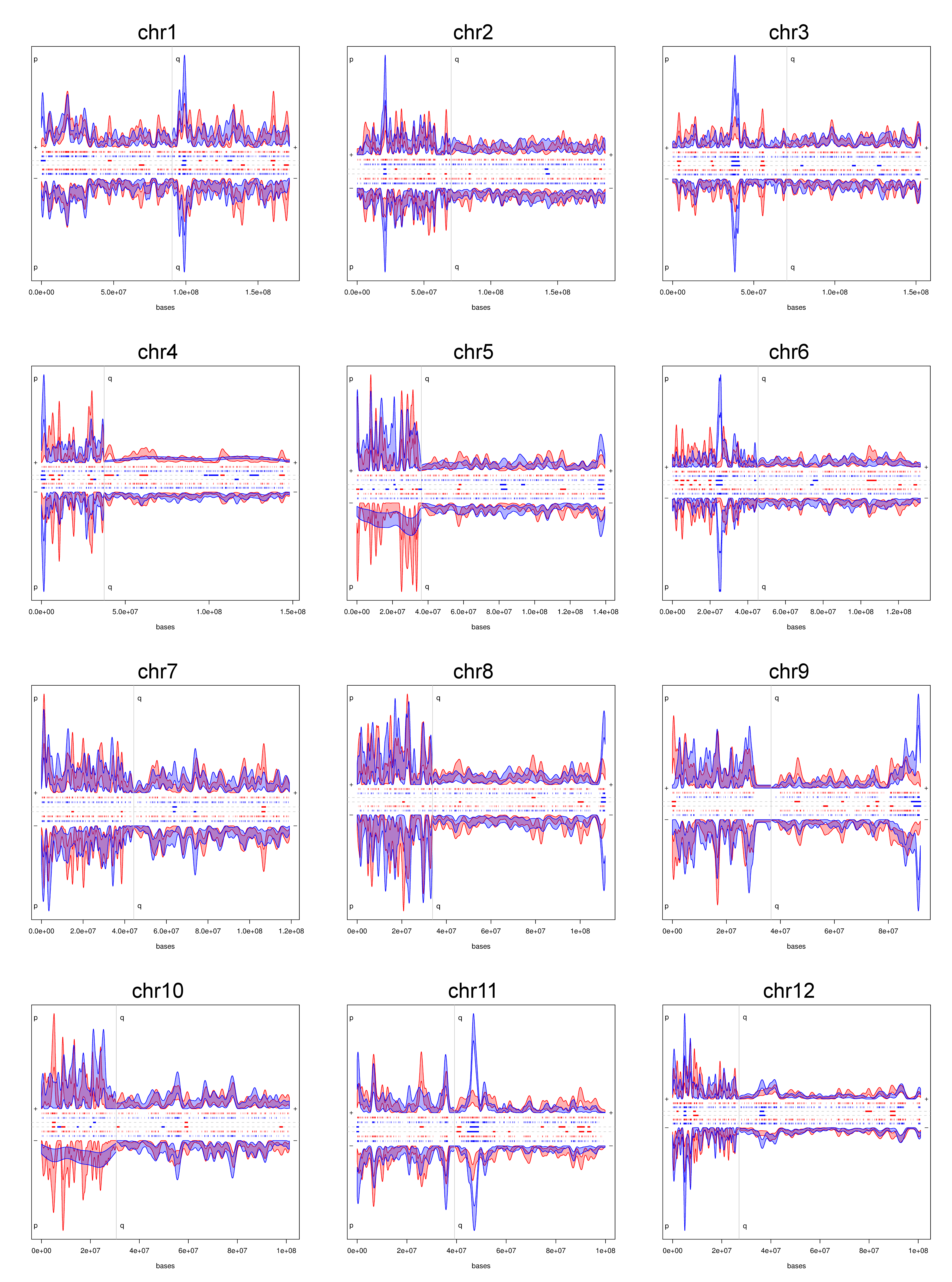

Supplement: Figure S1 — Integration densities of HIV and MLV in CD34+ cells, for chromosomes chr1, chr2, chr3, chr4, chr5, chr6, chr7, chr8, chr9, chr10, chr11 and chr12. We analyzed each strand separately: the upper half is the+strand and the lower the−strand. In blue the estimated variability band at level 0.99 for HIV integrations, in red for MLV. Candidate comparative hotspots are plotted in the two central x-axes, the color indicating which of the two vectors had stronger integration intensity (HIV: blue; MLV: red). In the other four x-axes, each tick represents one integration site, with the same color code. Because of resolution, many ticks fall on the same point and cannot be distinguished. (TIFF) [file pcbi.1002292.s001.tiff]

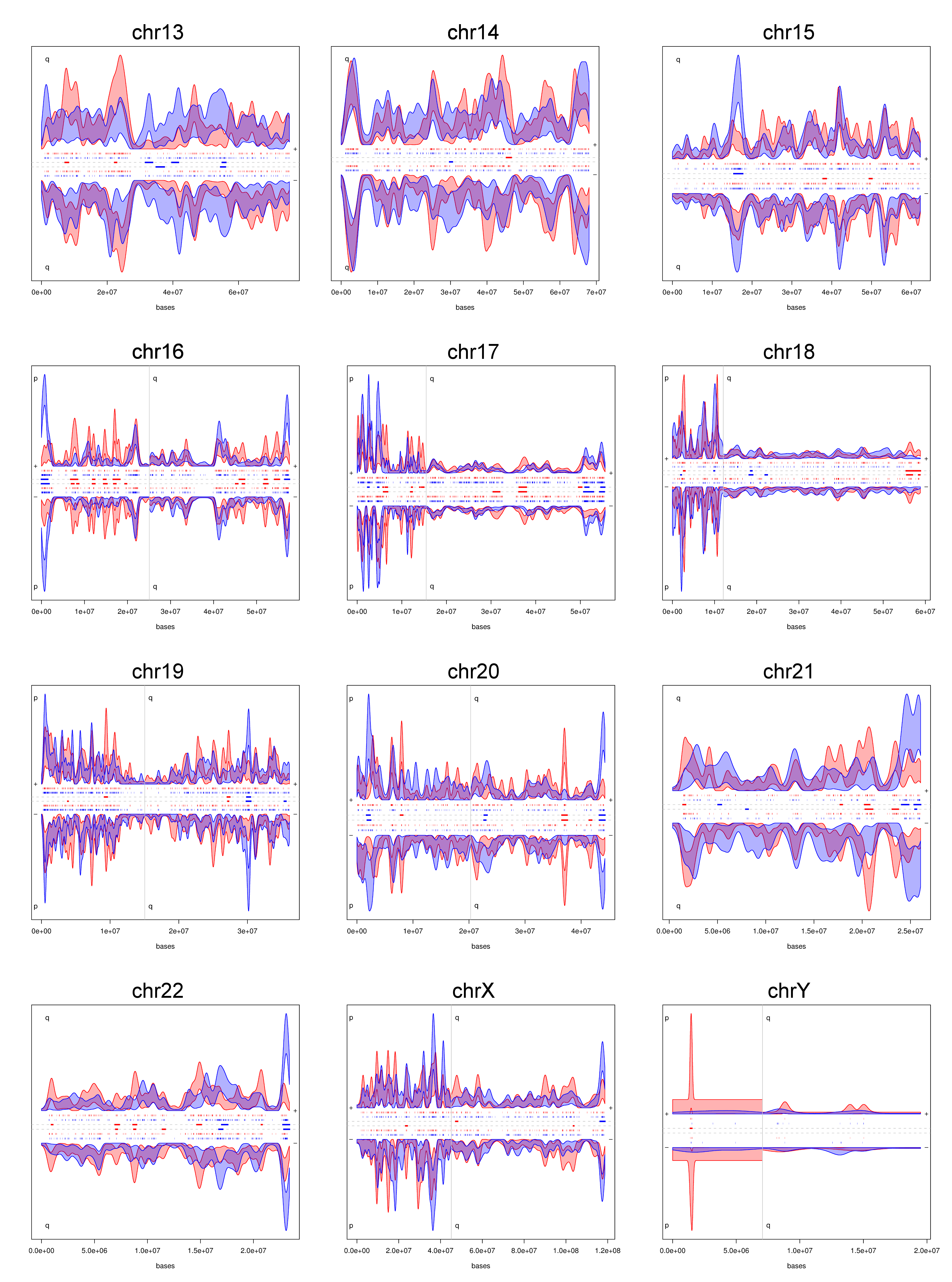

Supplement: Figure S2 — Integration densities of HIV and MLV in CD34+ cells, for chromosomes chr13, chr14, chr15, chr16, chr17, chr18, chr19, chr20, chr21, chr22, chrX and chrY. We analyzed each strand separately: the upper half is the+strand and the lower the−strand. In blue the estimated variability band at level 0.99 for HIV integrations, in red for MLV. Candidate comparative hotspots are plotted in the two central x-axes, the color indicating which of the two vectors had stronger integration intensity (HIV: blue; MLV: red). In the other four x-axes, each tick represents one integration site, with the same color code. Because of resolution, many ticks fall on the same point and cannot be distinguished. Since no integration was found in p-arm of chromosomes chr13, chr14, chr15, chr21 and chr22, in such cases only the q-arm was plotted. (TIFF) [file pcbi.1002292.s002.tiff]

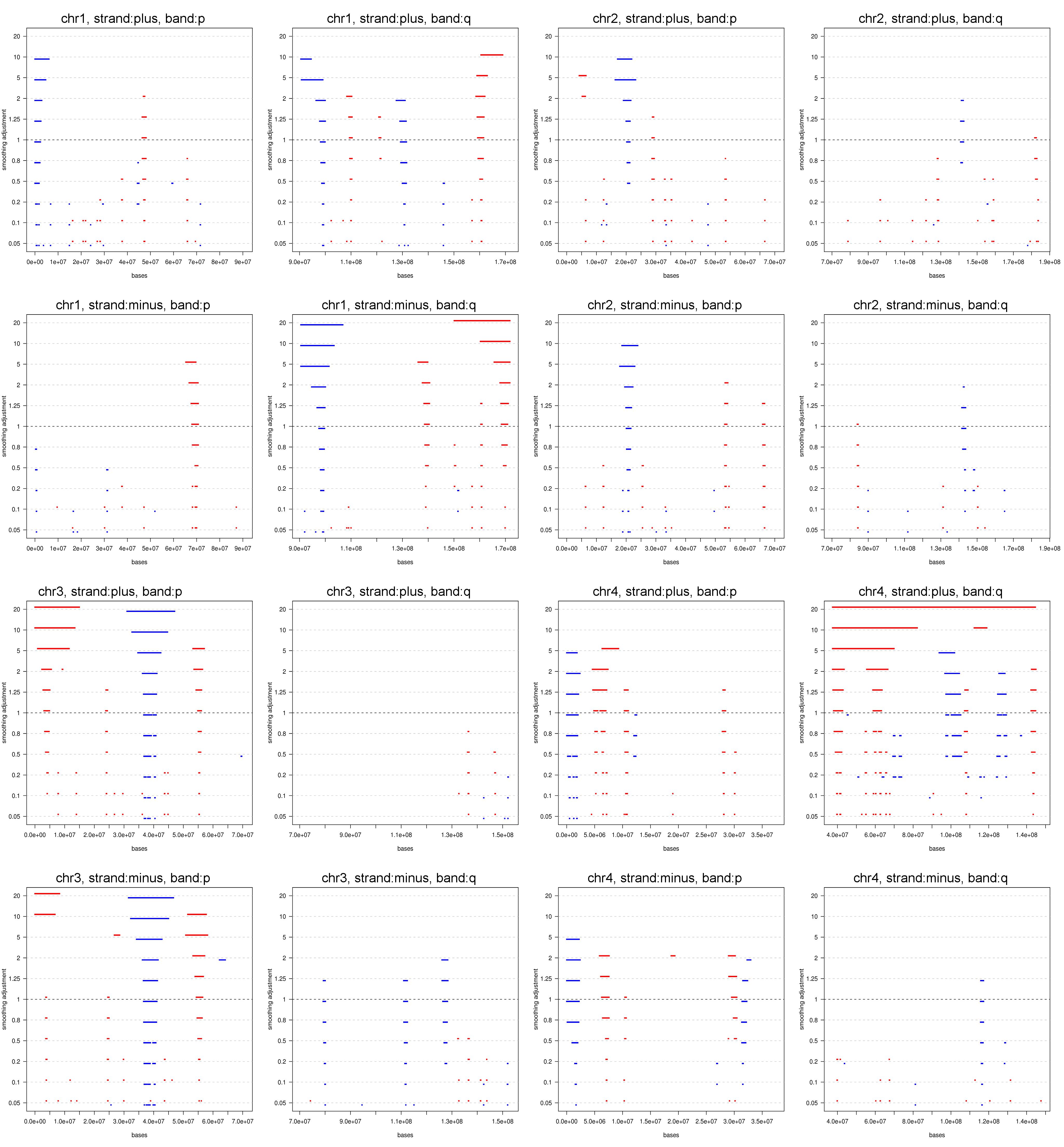

Supplement: Figure S3 — Robustness plots for all hotspots, chromosomes chr1, chr2, chr3 and chr4. The name of each figure identifies the chromosome and the arm and strand. In these figures we can observe how hotspots would change in length and location if we were to use different smoothing parameters. Most importantly, we see that the hotspots identified at level 1, corresponding to our choice of the smoothing parameters, persist at slightly larger and smaller values, confirming their validity. At smaller levels of smoothing many spurious hotspots appear, of very short length. There is no support from the data for these, as they either disappear for more smoothing or they merge into larger and more robust segments. (TIFF) [file pcbi.1002292.s003.tiff]

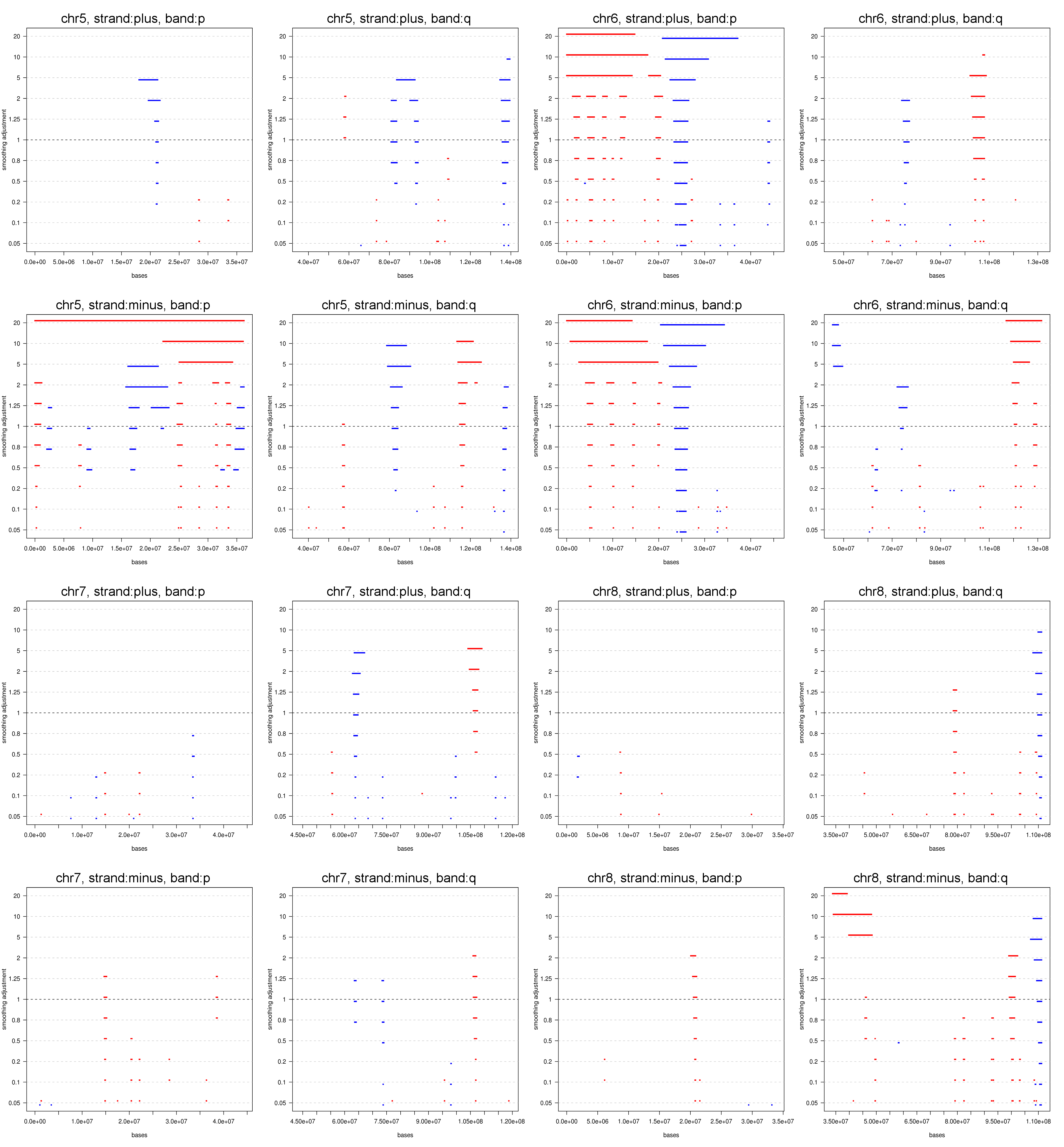

Supplement: Figure S4 — Robustness plots for all hotspots, chromosomes chr5, chr6, chr7 and chr8. The name of each figure identifies the chromosome and the arm and strand. In these figures we can observe how hotspots would change in length and location if we were to use different smoothing parameters. Most importantly, we see that the hotspots identified at level 1, corresponding to our choice of the smoothing parameters, persist at slightly larger and smaller values, confirming their validity. At smaller levels of smoothing many spurious hotspots appear, of very short length. There is no support from the data for these, as they either disappear for more smoothing or they merge into larger and more robust segments. (TIFF) [file pcbi.1002292.s004.tiff]

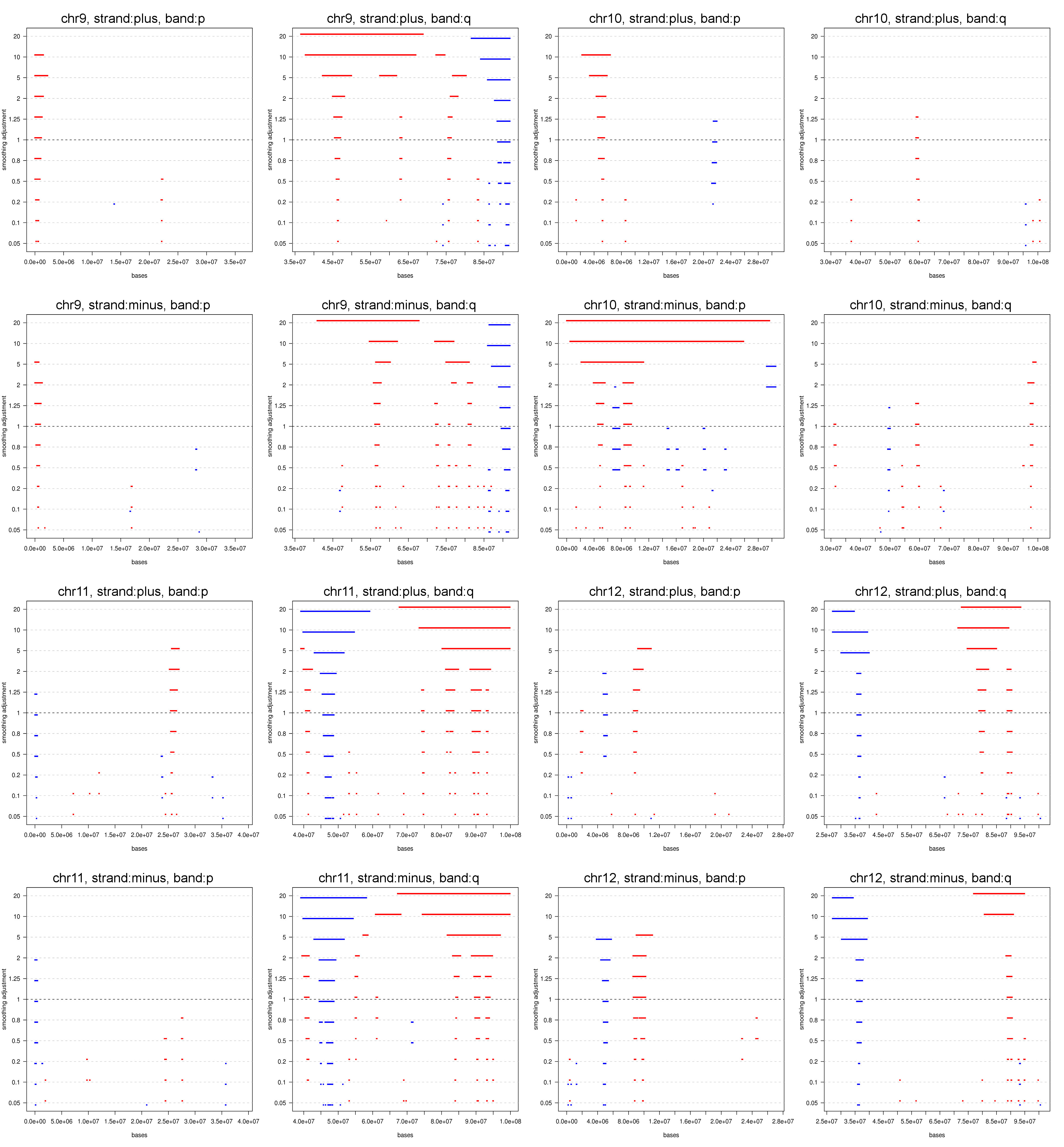

Supplement: Figure S5 — Robustness plots for all hotspots, chromosomes chr9, chr10, chr11 and chr12. The name of each figure identifies the chromosome and the arm and strand. In these figures we can observe how hotspots would change in length and location if we were to use different smoothing parameters. Most importantly, we see that the hotspots identified at level 1, corresponding to our choice of the smoothing parameters, persist at slightly larger and smaller values, confirming their validity. At smaller levels of smoothing many spurious hotspots appear, of very short length. There is no support from the data for these, as they either disappear for more smoothing or they merge into larger and more robust segments. (TIFF) [file pcbi.1002292.s005.tiff]

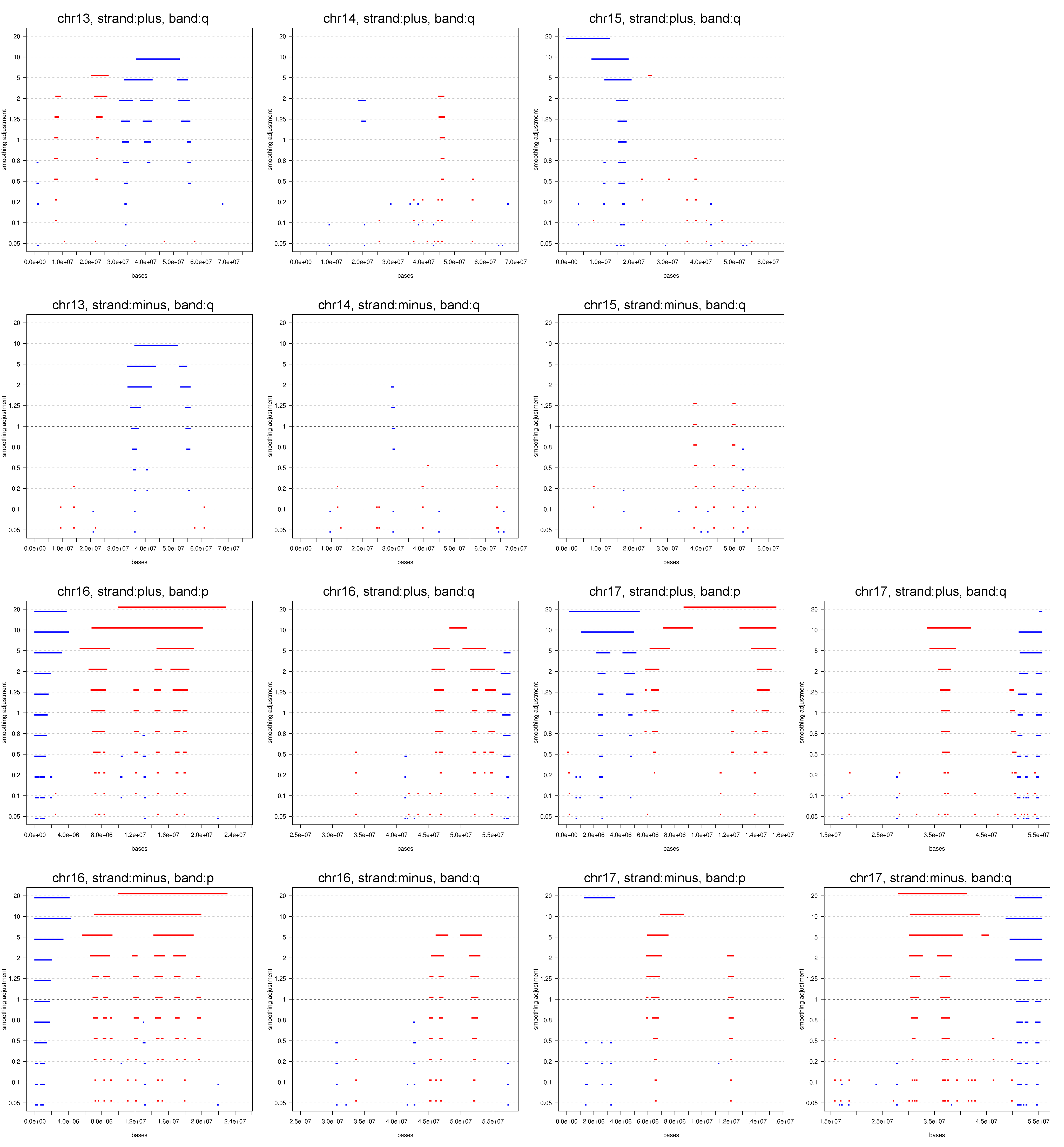

Supplement: Figure S6 — Robustness plots for all hotspots, chromosomes chr13, chr14, chr15 chr16 and chr17. The name of each figure identifies the chromosome and the arm and strand. In these figures we can observe how hotspots would change in length and location if we were to use different smoothing parameters. Most importantly, we see that the hotspots identified at level 1, corresponding to our choice of the smoothing parameters, persist at slightly larger and smaller values, confirming their validity. At smaller levels of smoothing many spurious hotspots appear, of very short length. There is no support from the data for these, as they either disappear for more smoothing or they merge into larger and more robust segments. Since no integration was found in p-arm of chromosomes chr13, chr14 and chr15 in such cases only the q-arm was plotted. (TIFF) [file pcbi.1002292.s006.tiff]

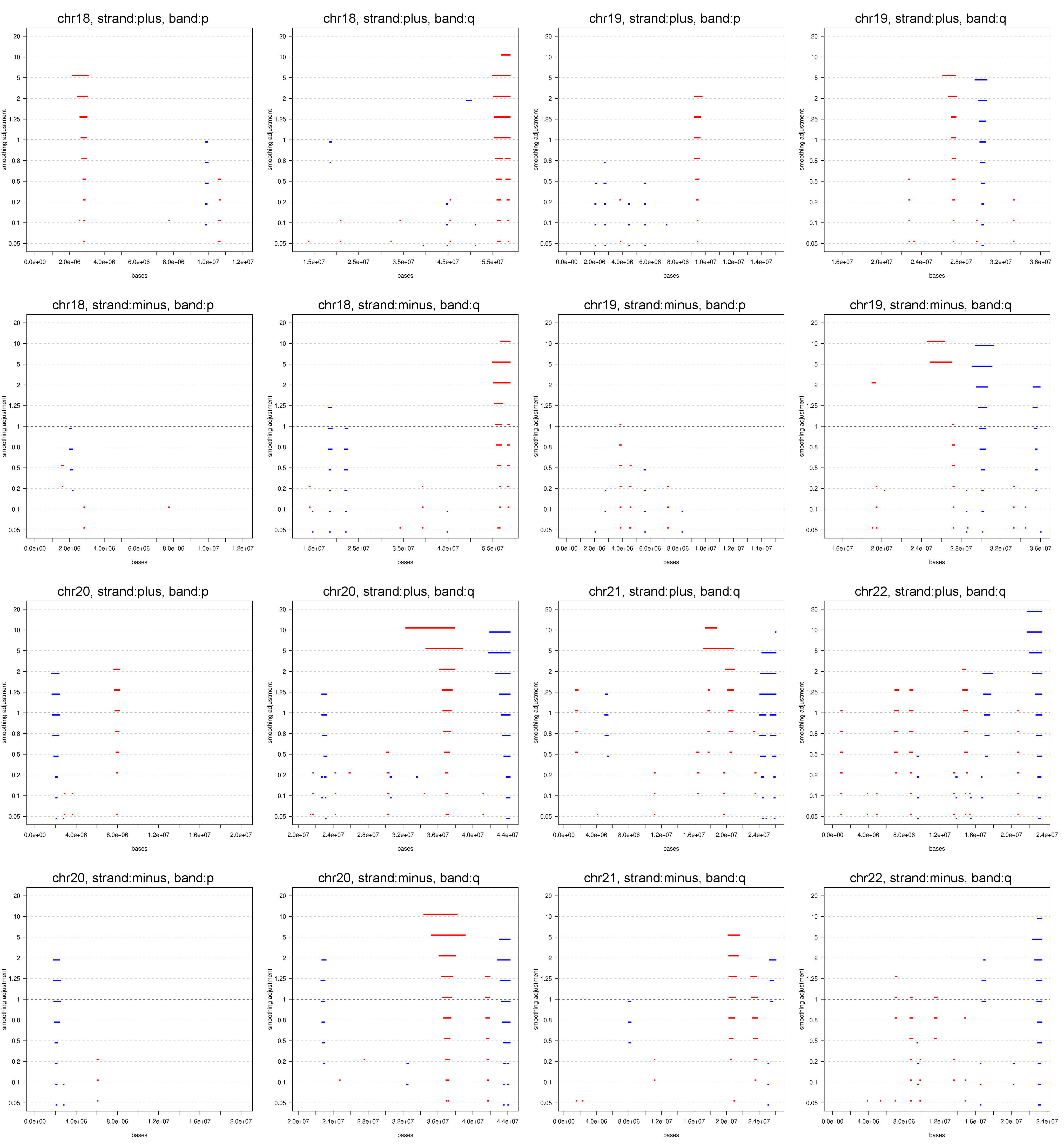

Supplement: Figure S7 — Robustness plots for all hotspots, chromosomes chr18, chr19, chr20, chr21 and 22. The name of each figure identifies the chromosome and the arm and strand. In these figures we can observe how hotspots would change in length and location if we were to use different smoothing parameters. Most importantly, we see that the hotspots identified at level 1, corresponding to our choice of the smoothing parameters, persist at slightly larger and smaller values, confirming their validity. At smaller levels of smoothing many spurious hotspots appear, of very short length. There is no support from the data for these, as they either disappear for more smoothing or they merge into larger and more robust segments. Since no integration was found in p-arm of chromosomes chr21 and 22 in such case only the q-arm was plotted. (TIFF) [file pcbi.1002292.s007.tiff]

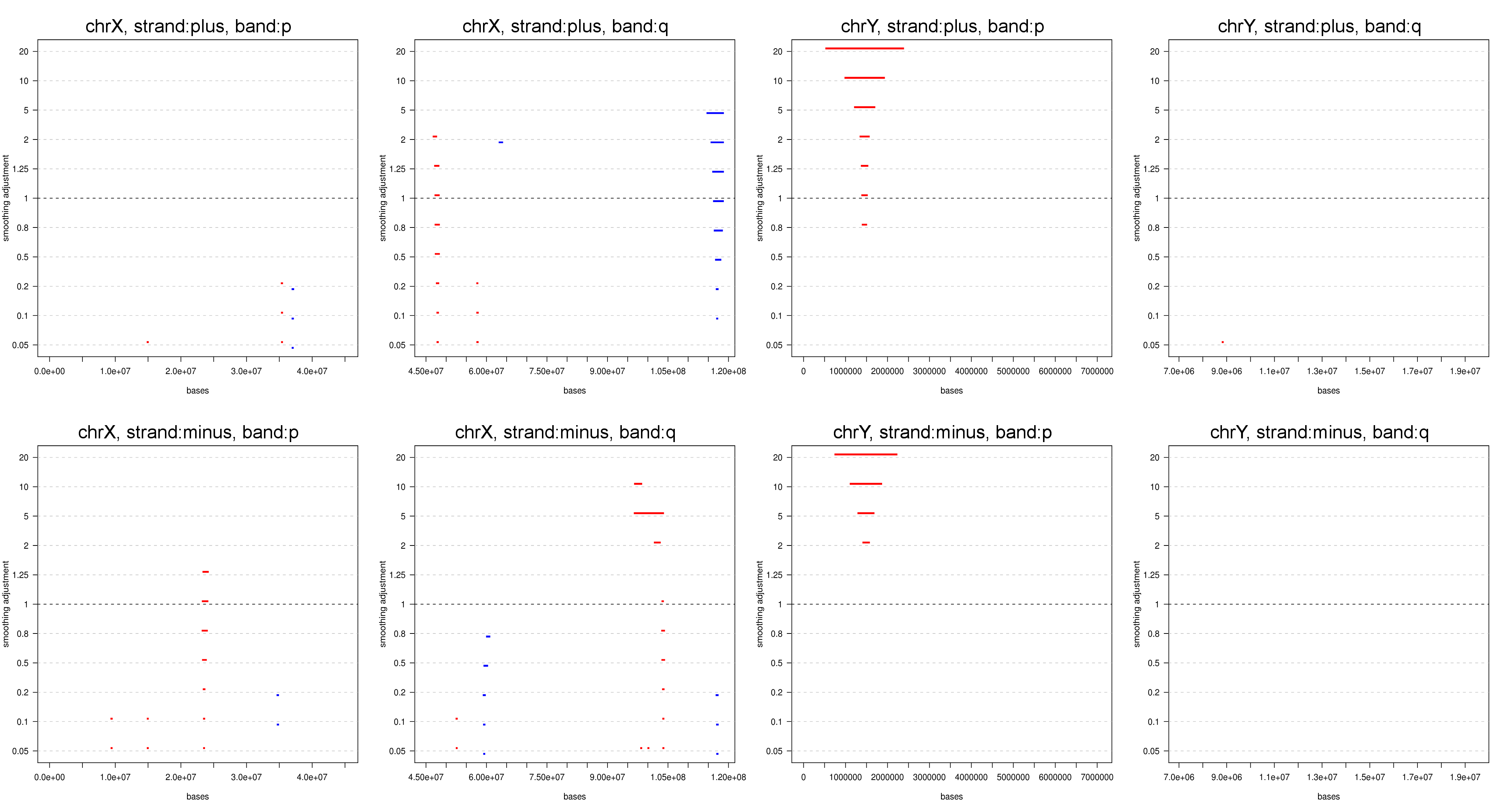

Supplement: Figure S8 — Robustness plots for all hotspots, chromosomes chrX and chrY. The name of each figure identifies the chromosome and the arm and strand. In these figures we can observe how hotspots would change in length and location if we were to use different smoothing parameters. Most importantly, we see that the hotspots identified at level 1, corresponding to our choice of the smoothing parameters, persist at slightly larger and smaller values, confirming their validity. At smaller levels of smoothing many spurious hotspots appear, of very short length. There is no support from the data for these, as they either disappear for more smoothing or they merge into larger and more robust segments. (TIFF) [file pcbi.1002292.s008.tiff]
