# Supplementary material for: Estimated Comparative Integration Hotspots Identify Different Behaviors of Retroviral Gene Transfer Vectors
Source: PLoS Comput Biol. 2011 Dec 1;7(12):e1002292. doi: 10.1371/journal.pcbi.1002292 (PMC3228801; doi:10.1371/journal.pcbi.1002292)
Supplement: Table S2 — Gene Ontology (GO) analysis of genes targeted by HIV and MLV comparative hotspots. 2027 and 475 genes targeted by HIV and MLV comparative hotspots were analyzed by the DAVID Functional Annotation tool 1,2, using the Human Genome as a background population. The table summarizes the significantly over-represented GO categories (GO terms) in the two datasets, after Bonferroni correction for multiple testing. The number of genes included in each GO category is specified (Count), together with their percentage (%) with respect to the total number of genes in the list (List Total) and the fold enrichment over the background. The GO class to which each category belongs is also given (BP: biological process, MF: molecular function, CC: cellular compartment). (DOC) [file pcbi.1002292.s010.doc]

**Supplemental Table 2. Gene Ontology (GO) analysis of genes targeted by HIV and MLV comparative hotspots.** 2027 and 475 genes targeted by HIV and MLV comparative hotspots were analyzed by the DAVID Functional Annotation tool[1,2], using the Human Genome as a background population. The table summarizes the significantly over-represented GO categories (GO terms) in the two datasets, after Bonferroni correction for multiple testing. The number of genes included in each GO category is specified (Count), together with their percentage (%) with respect to the total number of genes in the list (List Total) and the fold enrichment over the background. The GO class to which each category belongs is also given (BP: biological process, MF: molecular function, CC: cellular compartment).

| **HIV target genes (n=2027)** | |  |  |  |  |  |
| --- | --- | --- | --- | --- | --- | --- |
| GO class | GO Term | Count | List Total | % | Fold Enrichment | p-value  (Bonferroni corrected) |
| BP | GO:0019882~antigen processing and presentation | 26 | 1388 | 1.30 | 3.19 | 7.14E-04 |
| BP | GO:0048002~antigen processing and presentation of peptide antigen | 13 | 1388 | 0.65 | 4.72 | 1.83E-02 |
| BP | GO:0002474~antigen processing and presentation of peptide antigen via MHC class I | 10 | 1388 | 0.50 | 5.98 | 3.36E-02 |
| CC | GO:0042611~MHC protein complex | 21 | 1583 | 1.05 | 3.70 | 1.59E-04 |
| CC | GO:0005829~cytosol | 186 | 1583 | 9.30 | 1.41 | 5.31E-04 |
| CC | GO:0042824~MHC class I peptide loading complex | 8 | 1583 | 0.40 | 8.93 | 1.75E-03 |
| CC | GO:0042825~TAP complex | 7 | 1583 | 0.35 | 10.05 | 3.76E-03 |
| CC | GO:0016600~flotillin complex | 7 | 1583 | 0.35 | 7.82 | 3.72E-02 |
| CC | GO:0005737~cytoplasm | 801 | 1583 | 40.03 | 1.10 | 4.21E-02 |
| MF | GO:0005515~protein binding | 889 | 1491 | 44.43 | 1.11 | 1.90E-03 |
| MF | GO:0004886~retinoid-X receptor activity | 8 | 1491 | 0.40 | 9.03 | 3.33E-03 |
| MF | GO:0051059~NF-kappaB binding | 13 | 1491 | 0.65 | 4.55 | 1.04E-02 |
| MF | GO:0005488~binding | 1291 | 1491 | 64.52 | 1.05 | 1.72E-02 |
| MF | GO:0010861~thyroid hormone receptor activator activity | 8 | 1491 | 0.40 | 7.39 | 2.53E-02 |
| MF | GO:0030375~thyroid hormone receptor coactivator activity | 8 | 1491 | 0.40 | 7.39 | 2.53E-02 |
| MF | GO:0032395~MHC class II receptor activity | 10 | 1491 | 0.50 | 5.35 | 3.85E-02 |
|  | |  |  |  |  |  |
| **MLV target genes (n=475)** | |  |  |  |  |  |
| GO class | GO Term | Count | List Total | % | Fold Enrichment | p-value (Bonferroni correction) |
| BP | GO:0002455~humoral immune response mediated by circulating immunoglobulin | 9 | 341 | 1.90 | 12.02 | 1.18E-03 |
| BP | GO:0065007~biological regulation | 224 | 341 | 47.36 | 1.24 | 3.14E-03 |
| BP | GO:0007606~sensory perception of chemical stimulus | 31 | 341 | 6.55 | 2.68 | 3.89E-03 |
| BP | GO:0002250~adaptive immune response | 12 | 341 | 2.54 | 6.45 | 5.04E-03 |
| BP | GO:0002460~adaptive immune response based on somatic recombination of immune receptors built from immunoglobulin superfamily domains | 12 | 341 | 2.54 | 6.45 | 5.04E-03 |
| BP | GO:0006958~complement activation, classical pathway | 8 | 341 | 1.69 | 11.42 | 1.01E-02 |
| BP | GO:0050909~sensory perception of taste | 9 | 341 | 1.90 | 8.47 | 2.00E-02 |
| BP | GO:0050789~regulation of biological process | 211 | 341 | 44.61 | 1.23 | 2.69E-02 |
| BP | GO:0050890~cognition | 44 | 341 | 9.30 | 2.00 | 3.74E-02 |
| BP | GO:0002682~regulation of immune system process | 25 | 341 | 5.29 | 2.69 | 4.78E-02 |
| MF | GO:0004888~transmembrane receptor activity | 63 | 346 | 13.32 | 2.11 | 1.11E-05 |
| MF | GO:0004872~receptor activity | 72 | 346 | 15.22 | 1.71 | 2.78E-03 |
| MF | GO:0060089~molecular transducer activity | 82 | 346 | 17.34 | 1.58 | 1.01E-02 |
| MF | GO:0004871~signal transducer activity | 82 | 346 | 17.34 | 1.58 | 1.01E-02 |
| MF | GO:0004930~G-protein coupled receptor activity | 40 | 346 | 8.46 | 2.01 | 2.57E-02 |

1. Dennis G, Jr., Sherman BT, Hosack DA*, et al.* (2003) DAVID: Database for Annotation, Visualization, and Integrated Discovery. *Genome Biol* **4**: P3.

2. Huang da W, Sherman BT, Lempicki RA (2009) Systematic and integrative analysis of large gene lists using DAVID bioinformatics resources. *Nat Protoc* **4**: 44-57.
